# Rac1 Is Required for Epithelial Stem Cell Function during Dermal and Oral Mucosal Wound Healing but Not for Tissue Homeostasis in Mice

**DOI:** 10.1371/journal.pone.0010503

**Published:** 2010-05-06

**Authors:** Rogerio M. Castilho, Cristiane H. Squarize, Kantima Leelahavanichkul, Yi Zheng, Thomas Bugge, J. Silvio Gutkind

**Affiliations:** 1 Oral and Pharyngeal Cancer Branch, National Institute of Dental and Craniofacial Research, National Institutes of Health, Bethesda, Maryland, United States of America; 2 Division of Experimental Hematology, Children's Hospital Medical Center, University of Cincinnati, Cincinnati, Ohio, United States of America; 3 Department of Periodontics and Oral Medicine, University of Michigan, Ann Arbor, Michigan, United States of America; Leiden University, Netherlands

## Abstract

**Background:**

The regenerative capacity of the skin, including the continuous replacement of exfoliated cells and healing of injuries relies on the epidermal stem cells and their immediate cell descendants. The relative contribution of the hair follicle stem cells and the interfollicular stem cells to dermal wound healing is an area of active investigation. Recent studies have revealed that the small GTPase Rac1, which regulates cell migration and nuclear gene expression, is required for hair follicle stem function but not for the normal homeostasis of the interfollicular skin.

**Methodology/Principal Findings:**

Here we explored whether Rac1 contributes to wound healing in the skin and in the oral mucosa, the latter an anatomical site that presents similar architecture to that of the skin but is devoid of any hair follicle structures, and hence lacks hair follicle stem cells. Epidermal *Rac1* gene excision led to the clearly delayed closure of cutaneous wounds. Remarkably, genetic ablation of *Rac1* from the oral mucosa resulted in the complete inability of oral wounds to heal. We present evidence that the lack of oral mucosal re-epithelization may result from the reduced migratory capacity of cells lacking Rac1 together with altered expression of injury-induced proliferative and cellular stress-related expression programs.

**Conclusions/Significance:**

Together, these observations support that while the normal development and homeostasis of the interfollicular skin and oral mucosa do not require Rac1 function, the interfollicular and oral epithelial stem cells may require a Rac1-dependent program to orchestrate the tissue response to injury and ultimate for wound closure. Ultimately, these findings may enable the molecular characterization of the acute tissue regenerative response of these stem cell populations, thus facilitating the identification of novel molecular-targeted strategies aimed at accelerating wound closure.

## Introduction

The human epidermis is a remarkable organ involved in multiple vital functions, ranging from acting as a barrier that prevents the human body from loosing water, to protecting from contaminations by toxic chemicals and infections by microorganisms [1] [2] [3] [4]. These key functions require the integrity of the epidermal barrier. In the event of an injury, the fast response of the epidermis and its epidermal stem cells are required to reestablish local homeostasis and the epidermal barrier function [5] [6] [7] [4]. The ability to heal wounds relies on the rapid migration and proliferation of epithelial cells located in the basal layer of the epidermis adjacent to the area of tissue damage, and their ability to initiate terminal differentiation programs that result in the replenishment of the spinous, granular, and ultimately the cornified layers of the stratified epithelium [2] [7].

The regenerative capacity of the skin, including the continuous replacement of exfoliated cells and the restoration of tissue homeostasis upon injury relies on the epidermal stem cells, which possess self-renewal capacity, and by their immediate descendants, the transient amplifying cells, which divide a limited number of times and then undergo terminal differentiation [8]. While stem cells from the hair follicles (HF) can contribute to the healing process by migrating into the wound field to help reestablishing the epithelial barrier [9] [10] [11], an emerging body of evidence suggests that the interfollicular (IF) stem cells and their derived transient amplifying cells play a primary role in epidermal homeostasis and wound repair [12],[9] [10] [11]. However, the relative contribution of IF and HF stem cells to dermal wound healing is an area of active investigation, with recent studies supporting the emerging view that IF stem cells may be alone sufficient for the healing of cutaneous wounds [13].

In this regard, we have recently observed that the expression of the small GTPase Rac1 may distinguish HF from IF epithelial stem cell populations, as conditional deletion of Rac1 in the skin leads to the formation of rudimentary hair follicles showing reduction in length and absence of hair bulb structures without observing obvious phenotypes in the IF compartment [14]. Rac1 is a member of the Rho family of small GTPases involved in the regulation of multiple cellular functions, including cell migration, proliferation, and survival, by virtue of its ability to regulate the actin-based cytoskeleton and nuclear gene expression (reviewed in [15]). Rac1 has been implicated in cell-cell adhesion [16], in the maintenance of hair follicle stem cells [14] [17], and stem cell fate [18].

Whether Rac1 is required for normal tissue regeneration during the healing of dermal wounds in not fully understood. It was recently shown that the expression of a dominant inhibitory mutant of Rac1 delays epidermal wound-healing [19]. In these studies, excisional wound healing of the skin was also delayed by conditional deletion of the *Rac1* gene [19]. Nonetheless, the ability of the IF epidermis to respond to an injury in the absence of *Rac1* is still unclear, as large excisional wounds are highly dependent on the regenerative capacity of the HF stem cells [20], contrasting with recent observations that HF stem cells are dispensable for other more frequently encountered wound healing situations [13].

These observations prompted us to explore in detail the contribution of Rac1 to wound healing in the skin and in the oral mucosa, the latter an anatomical site that presents similar architecture to that of the skin but is devoid of any HF structures, and hence lacks HF stem cells. Using the Cre-lox technology, we conditionally excised the *Rac1* gene from the cytokeratin 14 (K14) expressing basal layer of the skin epidermis and oral mucosa. We show here that *Rac1* gene excision leads to a clearly delayed closure of cutaneous wounds and remarkably, genetic ablation of *Rac1* from the oral mucosa resulted in a complete inability of oral wounds to respond to stress and orchestrate the healing process.

## Materials and Methods

### Experimental mice

This study was approved by the Animal Care and User Committee (ACUC), according to NIH animal study protocols approved by the ACUC, protocol 06-408, National Institute of Dental and Craniofacial Research (NIDCR), in compliance with the “Guide for the Care and Use of Laboratory Animals.” Animals were housed on 12-h light/dark cycles and received food, standard rodent chow, and water *ad libitum* in compliance with AAALAC guidelines. The animals were observed daily by the investigators and animal care staff. Any animals displaying signs of discomfort, wasting, ruffled hair coat, hunching, or other signs indicative of distress were treated appropriately to alleviate discomfort or euthanized if recommended by animal care staff or the facility veterinary. C57BL/6j *Rac1* ^loxP/loxP^ mice [21] were crossbred with K14-Cre mice [22] to derive the K14Cre*Rac1* ^F/F^ mice [14].

### Skin full thickness incision and preparation of sections for histology

Experiments were performed on K14Cre*Rac1* ^F/F^ mice and control littermates. Mice were anesthetized through mixture of oxygen and isoflurane inhalation (Forane – Baxter Health Care Corporation), the dorsal skin was shaved and surgically prepared with consecutive applications of 10% providone iodine scrub, and 70% isopropanol. The surgical site of 15 mm was outlined with a sterile surgical marker as a template. Full-thickness incisional wounds were created using a #21 scalpel. Mice were followed until the wounds were clinically closed (7–17 days) and the wound area was measured daily and analyzed by GraphPad Prism software (GraphPad Software, Inc.). Wound closure was expressed as percentage of open wounds. Mice were also sacrificed by CO_2_ euthanasia at the indicated times and wound samples were collected. Skin samples were obtained from transgenic mice and controls, fixed overnight in 4% paraformaldehyde and transferred to 70% ethanol before being processed and embedded in paraffin. Tissue sections were stained by hematoxylin and eosin (H&E) for histology, or processed for immunohistochemistry and immunofluorescence assays.

### Oral wound preparation

For oral wound healing assays, cohorts of mice including K14Cre*Rac1* ^F/F^ mice and control littermates were anesthetized using a mixture of oxygen and Isoflurane inhalation and secured with a nose cone. Using forceps, the tongue was tractioned to the opposite side of the surgical site of the mouth. The wound was inflicted using a 2 mm punch biopsy (Miltex, York, Inc. PA, USA) in the buccal mucosa anatomical site taking caution to avoid rupturing any major vessels by trans-illuminating the surgical site. Surgical dye was then applied to the wounded area to help the subsequent clinical and histological localization of the wound. The wounds were removed at the indicated days by dissecting the entire buccal mucosa followed by 4% paraformaldehyde fixation. Careful sagittal incision of the wounded area using the surgical dye as a guide was performed and used as a reference for histological sectioning.

### IHC, IF, antibodies, reagents, and immunoblotting

Immunohistochemistry (IHC), immunofluorescence (IF), and Western immunoblots (WB) were performed as previously described [14] [23]. Briefly, for IF, primary antibodies were incubated overnight and washed with PBS following by incubation with secondary antibodies conjugated to either fluorescein or rhodamine (Jackson ImmunoResearch Labs 1∶100) and mounted with media containing DAPI (Vector laboratories). The following antibodies were used at the specified dilutions: cytokeratin 10 (159P, Covance; 1∶1000 IF), cytokeratin 14 (155P, Covance; 1∶1000 IF), cytokeratin 6 (169P, Covance; 1∶500 IHC), fibrinogen/FITC (Dako; 1∶500 IF), GAPDH (FL-335, Santa Cruz; 1∶1000 WB), Rac1 (Upstate, WB), p-JNK (Cell Signaling; 1∶1000 WB), JNK (Cell Signaling; 1∶1000 WB), p-PAK1 (Cell Signaling; 1∶1000 WB), and PAK1 (Cell Signaling; 1∶1000 WB). Images were taken using either a Zeiss monochrome (MRm) or color (HRc) digital cameras attached to a Zeiss Axio Imager Z1 microscope equipped with an Apotome device (Carl Zeiss, Thornwood, NY).

### GST pull-down

Rac1 activity was assessed by glutathione S-transferase (GST) pull-downs, using the GST-PAK-CRIB (Cdc42/Rac interacting binding domain) bound to glutathione-Sepharose beads (Amersham Biosciences, General Electric's, Piscataway, NJ) as previously described [24]. Briefly, bacterially expressed GST fusion protein containing the CRIB domain of PAK1 was bound to glutathione-Sepharose beads. Cells were lysed with ice-cold buffer containing 10 mM Tris, 100 mM NaCl, 1% Triton X-100, 0.5 mM EDTA, 40 mM β-glycerophosphate, 10 mM MgCl_2_, 1 mM Na_3_VO_4_, 10 μg/ml aprotinin, 10 μg/ml leupeptin, and 1 mM phenylmethylsulfonyl fluoride and incubated for 30 min with purified beads. Active form of Rac1 associated with GST-CRIB was then quantified by WB using a Rac1 monoclonal antibody.

### Establishment of Normal Oral Keratinocytes Spontaneously Immortalized (NOK-SI)

Gingival tissues from healthy volunteers were obtained at the NIDCR clinic (Clinical Protocol # 06-D-0144). After surgery, fresh tissues derived from the retro molar area of the oral cavity were incubated overnight in trypsin 0.25% (Sigma Aldrich, CA) at 4°C. Next day, the epithelial compartment was mechanically dissociated from the connective tissue and minced to fine fragments. Keratinocytes were then filtered through a 100 µm cell strainer (BD falcon), pellet at 125 g for 5 min at 4°C, resuspended in keratinocyte serum-free culture medium (Invitrogen, CA), and plated on 60 mm dishes kept in a controlled humidity, temperature, and CO_2_ environment [25]. Using this procedure, we observed that most human oral epithelial cells can be expanded for a finite number of passages, achieving a replicative senescent state after approximately 55 days, but few cultures escaped cell senescence, and instead became immortal (G.L. Sanchez, K.L, R.C., J.S.G et *al*., manuscript in preparation). To date, these cells, termed spontaneously immortalized normal oral keratinocytes, NOK-SI, have been cultured for more than 2 years, retaining epithelial morphology, proliferative capacity, and the expression of typical markers such as cytokeratins and E-cadherin. NOK-SI cell line is routinely cultured in Keratinocyte-SFM medium (Gibco, USA) supplemented with BPE and EGF, penicillin, streptomycin, and maintained at 37°C in a 5% CO2-humidified incubator.

### Cell proliferation and migration

Cell proliferation was measured by [^3^H]-thymidine incorporation as described [26]. Directional cell migration of primary keratinocytes was studied in cell monolayers using an *in vitro* scratch wound assay. NOK-SI cells were seeded in confluence on 35 mm tissue culture dishes using a 200 µl disposable plastic pipette tip. The cell monolayer was scratched and cells were then allowed to migrate. The cells were observed every 2 h for 24 h and photographed using an Axiovert 200M microscope (Carl Zeiss, Germany).

### Rac1 knockdown

Cells were seeded in 24-well or 6-well plates and when they reached 70% confluence, the cells were washed twice in serum-free medium and transfected with a total 50 nM of double-stranded small interfering RNA (siRNA) directed against human *RAC1* (Qiagen, HS_RAC1_6, SI02655051 and Santa Cruz Biotechnology, SC-36351) using HyperFect (Qiagen) following the manufacturer's instructions. The optimal concentrations of siRNA and time points were determined by performing a dilution curve of siRNA for each sequence and the knockdown were determined by Western blot analysis. Human siRNA from Qiagen (siRNA#2) was selected for its ability to induce superior Rac1 down regulation and used thereafter. After 72 h of transfection, cells were treated as indicated. The sequences of the non-targeting control siRNA (Qiagen) oligonucleotides used as a control were as follows (5′-UUCUCCGAACGUGUCACGUdTdT-3′, and 5′-ACGUGACACGUUCGGAGAAdTdT-3′).

### Statistical analysis

Statistical analyses were performed by ANOVA analysis of variance test. The Kaplan-Meier analysis followed by log rank test was performed to analyze time to wound closure. Ki-67 staining was analyzed by using GraphPad Prism 4.03 (GraphPad Software, San Diego, CA). 3H incorporation was analyzed using one way ANOVA followed by Newmans-Keuls multiple comparison test. Asterisks denote statistic significance (NS, P>0.05; * P<0.05; ** P<0.01; and *** P<0.001).

## Results

### Conditional epidermal excision of the *Rac1* gene leads to a delay in cutaneous wound healing

**Figure 1:**
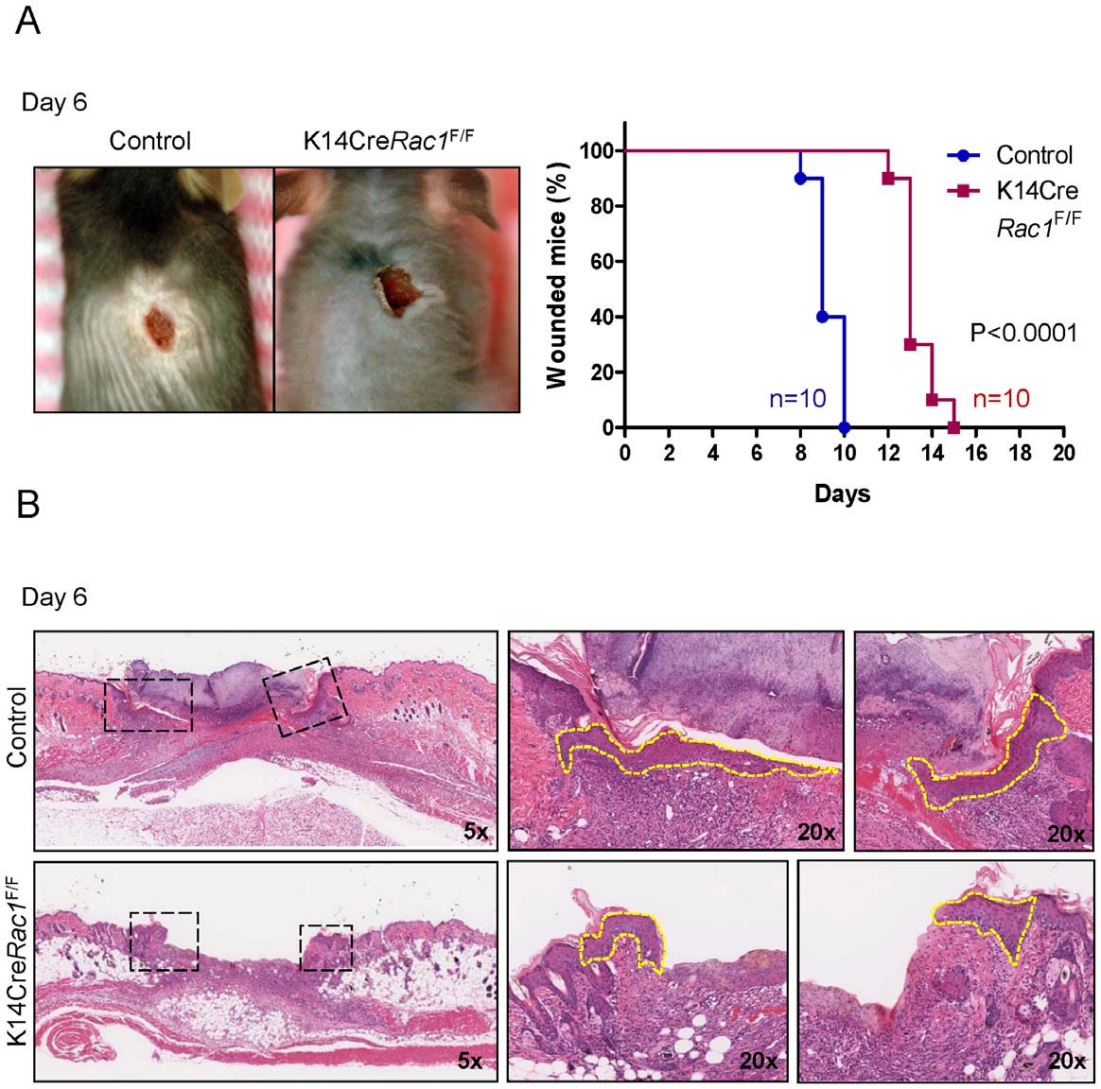
Defective epidermal wound-healing in *Rac1* deficient epidermis. (A) K14Cre*Rac1* ^F/F^ conditional knockout mice show delayed wound-healing, as shown on day 6 after injury compared with control littermates. There was a significant decrease in the rate of wound closure (p<0.0001) when comparing the percentage of mice exhibiting open wounds after surgical incision in K14Cre*Rac1* ^F/F^ mice (red squares, n  =  10) and control mice (blue circle, n  =  10) at the indicated days. (B) Left panels, representative H&E stained histological sections of wounded epidermis at day 6 after wounding showing the normal extension of the epithelial tongue of control (uppers panel) but defective epithelial tongue in K14Cre*Rac1* ^F/F^ mice (lower panel) showing limited migration at day 6. High magnification (20x) of the epithelial tongue of control and K14Cre*Rac1* ^F/F^ mice (dashed rectangles) are shown in the corresponding left panels. Epithelial tongues are delimitated in yellow dashes.

We have previously generated a conditional knockout mouse ablating the *Rac1* gene exclusively from the basal layer of the epidermis and oral epithelium [14], by crossing K14Cre^+/−^ with *Rac1* ^F/+^ and backcrossed with *Rac1* ^F/F^ to obtain K14Cre^+/−^ Rac1^F/F^ homozygous mouse line. After birth, conditional knockout homozygous mice lack expression of *Rac1* in the skin and the stratified epithelium lining the oral cavity, an absence of hair shafts but normal IF epidermis and oral mucosa [14]. Regarding the skin phenotype, similar results were also achieved by others using a related conditional epidermal *Rac1* knockout animal model, demonstrating the importance of Rac1 in the HF integrity but not for the development and homeostasis of the IF compartment [17]. Next, we decided to determine the *in vivo* effect of *Rac1* excision in these animals after the induction of a physiological stress on the epidermis through the infliction of a wound in the dorsal skin. For this purpose, we performed 15-mm-long full-thickness dermal wounds and followed the recovery of these wounds for over 20 days (Figure 1A). The lesions were examined by visual inspection and scored as healed when there was a complete loss of the wound crust followed by complete closure of the incision interface and re-establishment of the epidermal covering. K14Cre *Rac1* ^F/F^ mice showed a substantial delay in wound healing with a median healing time of 13 days compared to control mice that healed in 9 days (Figure 1A).

**Figure 2:**
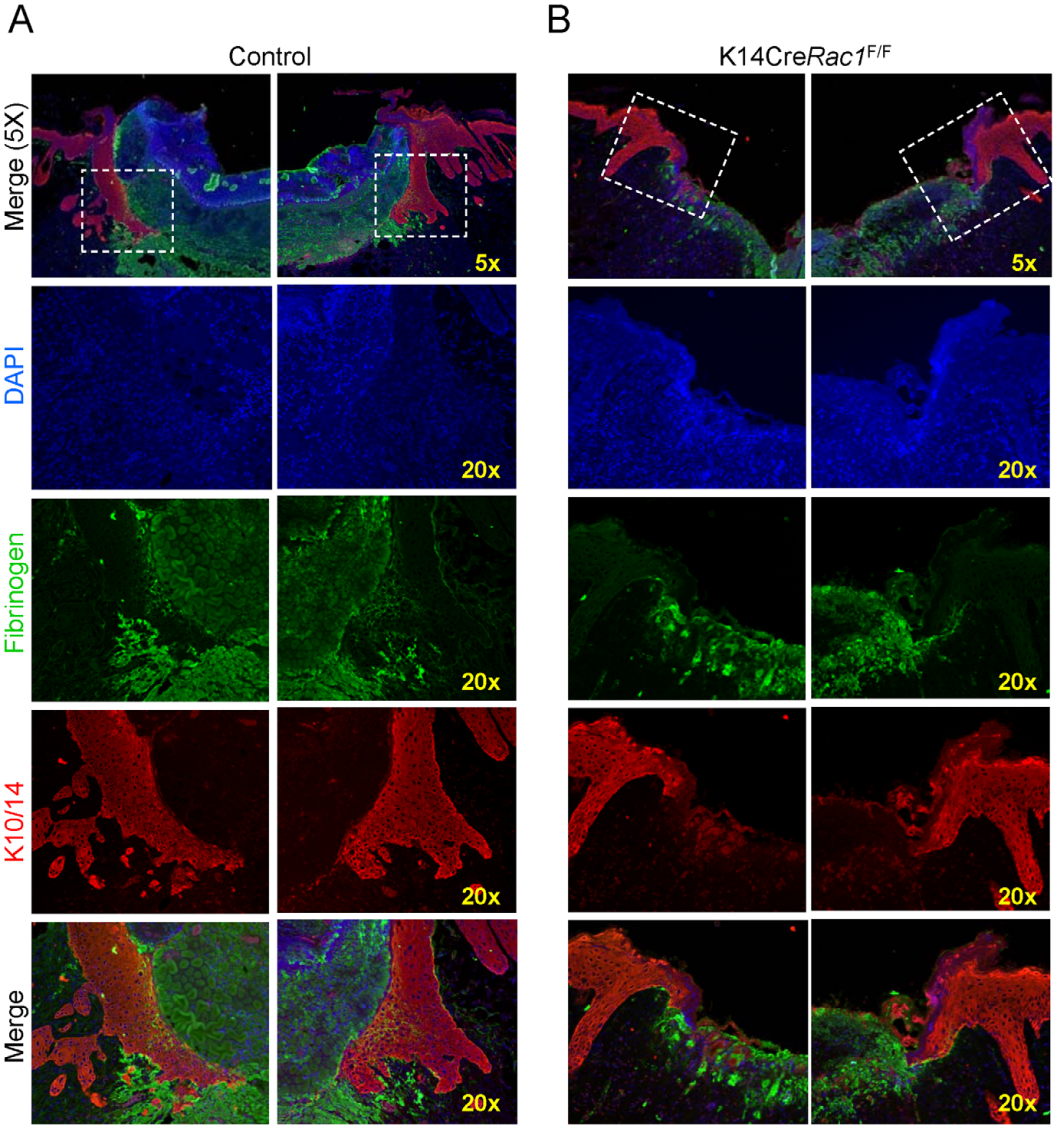
Deficient epithelial tongue migration in K14CreRac1^F/F^ mice. (A) Control mice. Immunofluorescence for cytokeratins 10 and 14 (K10/14, red) reveals the epidermal layer of the skin and the epithelial tongue migrating underneath the fibrin clot at day 6 (Green). DNA staining (Hoechst 33342-blue) delineates cellular nuclei. Note the extensive migration of the epithelial tongue in merged figure (high Magnification-20x). (B) K14Cre*Rac1* ^F/F^ conditional knockout mice stained for cytokeratins 10 and 14 (K10/14, red) show a more limited migration of the epithelial tongue by day 6 after injury. The deficient migration of the epithelial tongue is visualized in the merged figure (High magnification-20x).

During the process of wound healing, migrating keratinocytes form a thin wedge-shaped epithelial tongue that constitutes the leading wound edge. At the same time, there is an increase in the thickness of the spinous layer adjacent to wound and a rapid proliferation of epithelial cells immediately next to the migrating epithelial tongue edge [27]. Histological analysis of this process six days after incision revealed a dramatic reduction in the capacity of the epithelial tongue from K14Cre *Rac1* ^F/F^ mice to migrate into the wound bed (Figure 1B). We next used double immunofluorescence staining of tissue sections for fibrin(ogen) using FITC conjugated antibodies (green) and a mix of cytokeratins 10 and 14 (TRITC) (red) to visualize the fibrin(ogen) and keratinocytes simultaneously. This approach enabled the precise localization of the epithelial tongue of control mice, which dissects the necrotic tissue from the granulation tissue (Figure 2A), thus achieving a better resolution than H&E stained sections. This revealed that the migration of the epithelial tongue of K14Cre Rac1^F/F^ mice was greatly impaired after wounding when compared to control mice (Figure 2B).

### The *Rac1* gene is strictly required for the regeneration of murine oral mucosa

Recent findings suggest the necessity to consider the IF and HF stem cells as two distinct cell populations, which are capable of cooperatively repair the wounded epidermis [9]. Therefore, to specifically explore the contribution of Rac1 to wound closure mediated by IF stem cells, we decided to study the effect of *Rac1* excision in the oral mucosa, an epithelial tissue lacking any hair follicle appendages and their corresponding stem cells.

**Figure 3:**
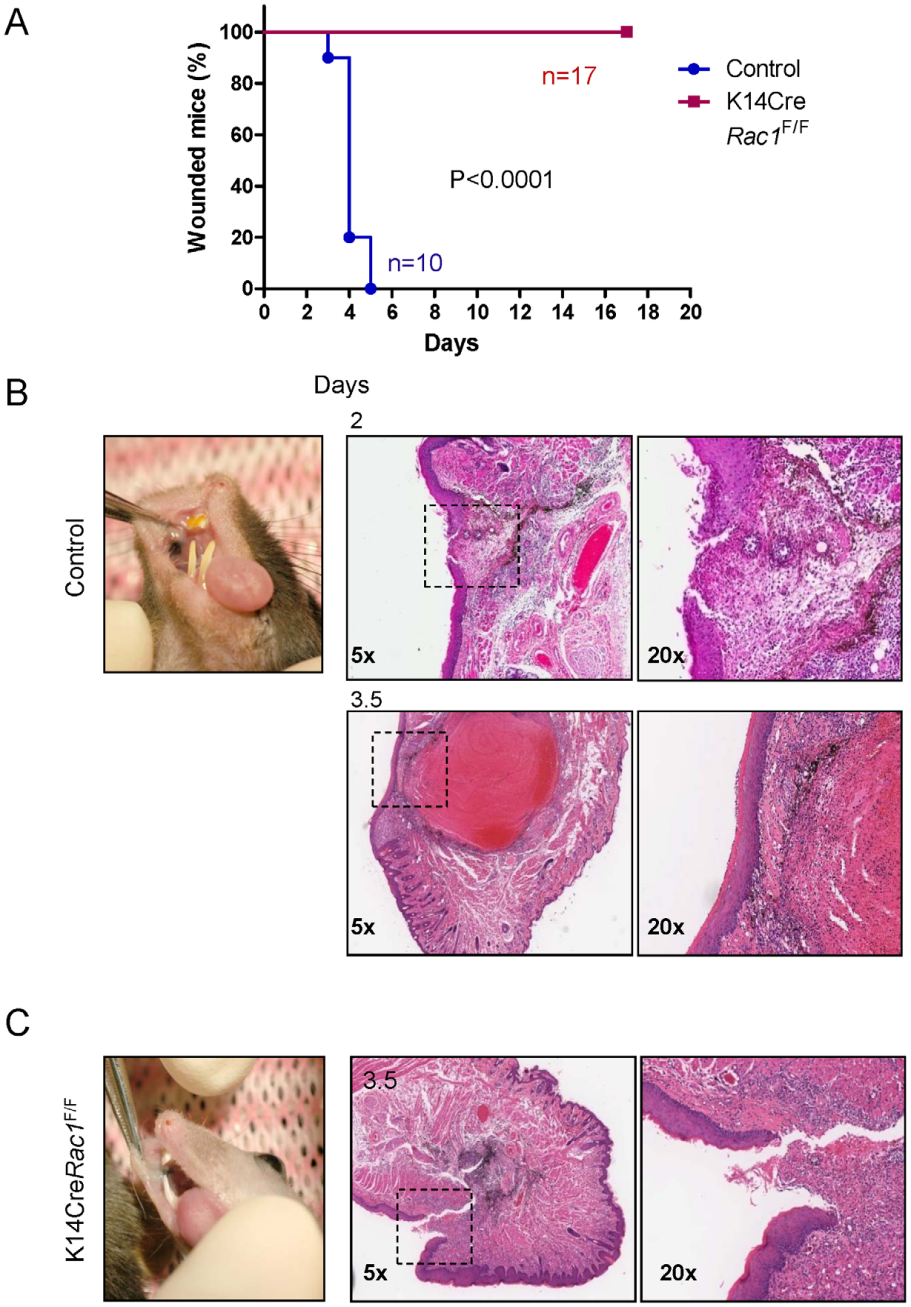
Absence of oral wound-healing in K14Cre*Rac1* ^F/F^ mice. (A) K14Cre*Rac1* ^F/F^ mice show a complete absence of oral wound closure when compared to control littermates (p<0.0001). The percentage of mice exhibiting open oral wounds after surgical incision in K14Cre*Rac1* ^F/F^ mice (red squares, n  =  17) and control mice (blue circle, n  =  10) are depicted at the indicated days. (B) left panel, representative control mouse showing the intraoral wound site stained with surgical dye (black spot) 3.5 days after injury. Middle panels, H&E stained sections of control mice 2 and 3.5 days after oral injury showing active epithelial tongue migration over the fibrin clot by day 2, and complete closure of the wound site by day 3.5. Right panels, higher magnification (20x) of the corresponding area depicted with dashed rectangles in the middle panels. (C) Left panel, K14Cre*Rac1* ^F/F^ mouse showing the intraoral surgical anatomical site stained in black (surgical dye) at day 3.5 after surgery. Middle panel, H&E staining of K14Cre*Rac1* ^F/F^ mice show lack of healing capacity of the oral mucosa with a complete absence of an epithelial tongue. Right panel, high magnification (20x) of the open wound depicted by a dashed rectangle in the middle panel.

We focused the oral wound-healing studies to the buccal mucosa, as this anatomical site provides easy access for punch biopsy intervention without impairing the feeding behavior of the mice. Using two millimeter biopsy punches, we performed a unilateral punch in K14Cre^+/−^ *Rac1* ^F/F^ and control mice. The median time for healing of control mice was about 3.5 days, however no healing signs could be detected by H&E analysis in the K14Cre *Rac1* ^F/F^ group at that time point. Next, we extended the observation of the K14Cre *Rac1* ^F/F^ group post surgery in the attempt to identify the day in which the wounds heal; however no histological signs of healing were detected when analyzed up to 17 days after wounds (Figure 3A and data not shown). We decided to compare the wounds from K14Cre *Rac1* ^F/F^ with two distinct control groups; the chronological control group by day 3.5 showing complete epithelial healing, and the physiological control group by day 2 showing open wounds with active epithelial tongue migration, thus resembling the status of the wounds of the K14Cre *Rac1* ^F/F^ mice.

**Figure 4:**
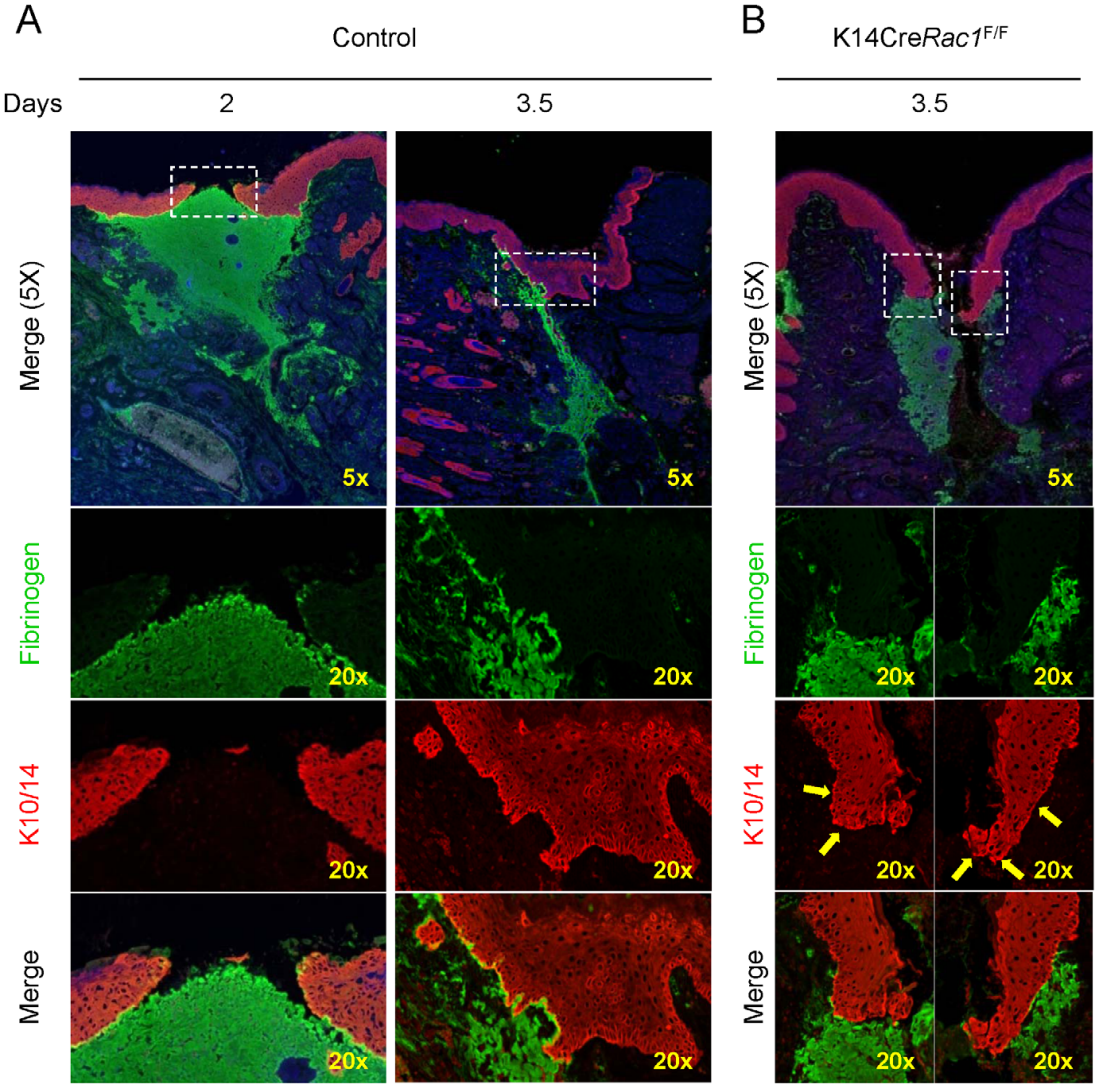
Lack of oral epithelial tongue migration in K14CreRac1^F/F^ mice. (A) Immunofluorescence for cytokeratins 10 and 14 (K10/14, red), fibrinogen (green) and DNA staining (Hoechst 33342-blue) delineating cellular nuclei of control tissue sections staining the oral mucosa. Two days after the oral wound the epithelial tongue is visible migrating over the fibrin(ogen) clot (green) and the oral mucosa is healed by day 3.5. (B) Tissue sections from K14Cre*Rac1* ^F/F^ mice stained as above show complete impairment of oral mucosa migration and maintenance of the punch biopsy trajectory evident by the fibrin(ogen) staining by day 3.5 (green). Note that high magnification (20x) of K10/14 staining show progressive loss of the well defined basal layer of the oral mucosa and absence of a epithelial tongue (yellow arrows).

Histological analysis revealed that control mice already exhibit advanced migration of the epithelial tongue over the granulation tissue by day 2, and by 3.5 days the wound site is totally healed (Figure 3B). In contrast, K14Cre Rac1^F/F^ mice still had open wounds by day 3.5 with the epithelial tongue showing no signs of epithelial migration into the wound area (Figure 3C). Of interest, by IF analysis, it was clear that the epithelial tongues in the control group migrate over the granulation tissue containing fibrin(ogen) 2 days post surgery, and achieve a complete closure of the oral wound already by day 3.5 (Figure 4A). We could observe an increased thickness of the regenerating epithelium, which is probably due to increased cellular proliferation concomitant with the presence of a well organized basal layer of the oral mucosa above the residual fibrin(ogen). The latter likely accumulated in the wound field during the initial clotting upon surgery. In contrasts, the epithelial tongues of the K14Cre*Rac1* ^F/F^ mice did not migrate by day 3.5, and present no signs of epithelial thickening. Morphologically, the cells next to the epithelial edge did not give rise to an epithelial tongue; rather the epithelial edge progressively loses its well defined basal cell layer morphology (Figure 4B, yellow arrows). While these morphological changes resemble the acquisition of a more differentiated phenotype at the edge of the wound, we could not detect the upregulated expression of cytokeratin 1 or loricrin in the basal layer (data not shown), which would have supported the activation of terminal differentiation programs.

### *Rac1* is required for human oral keratinocyte migration and proliferation

**Figure 5:**
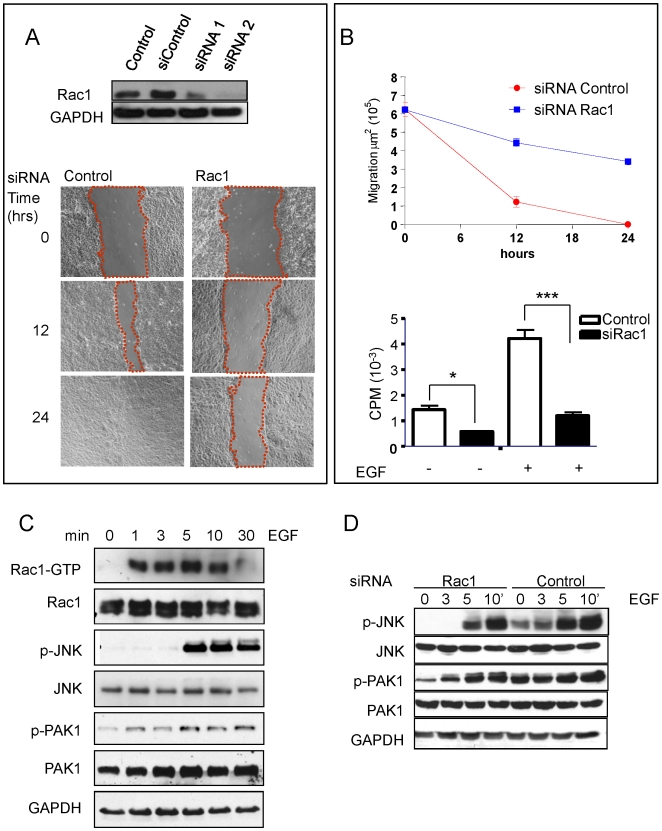
Impairment of human oral keratinocytes migration and proliferation after knock down of Rac1. (A) The human oral keratinocyte cell line (NOK-SI) was transfected with siRNAs and knockdown of Rac1 was confirmed by Western blot analyses of Rac1 in cellular lysates 72 h after transfection of two different targeting siRNAs (siRNA#1 and siRNA#2). A non-targeting siRNA oligonucleotide (siControl) and untransfected cells (Control) were used as controls. Scratch wound assay in NOK-SI cell line after control and Rac1 siRNA#2. Scratches were generated after cell confluence. *In vitro* cell migration and wound closure were assessed every 12 h. Representative pictures of the control and Rac1 siRNA#2 transfected cell cultures at the indicated time after the initial scratch are depicted. (B) Top graphic, areas of migration were measured (dotted line from figure A) in multiple wells and represented at the indicated time. The lower graphic shows cell proliferation of NOK-SI transfected with siRNA#2 against Rac1. Incorporation of [^3^H]thymidine in cells stimulated with EGF (+) (30 ng/ml) or left untreated (-) was measured by the accumulation of radioactivity in cellular DNA, and represented as the average of CPM± s.e.m. in triplicate samples from a representative experiment that was repeated three times (***p<0.001; *p<0.05). (C) GST pull-down of active Rac1 was performed using NOK-SI stimulated with EGF. Cell lysates were incubated with GST-PAK-N for 30 min to affinity precipitate active Rac1. PAK-bound Rac1 and total Rac1 in the corresponding total lysates were analyzed by Western blotting with a monoclonal antibody against Rac1. Total and phospho-specific antibodies were used to detect two downstream targets of Rac1, JNK and PAK1 and their corresponding phosphorylated species. (D) NOK-SI cells were transfected with Rac1 siRNA (siRNA#2) or control siRNA (siControl) and stimulated with EGF (30 ng/ml). Western blot analysis of the Rac1 downstream targets JNK and PAK1 show absence of basal p-JNK and 3 min after EGF stimulation compared to control that show detectable basal levels of p-JNK that increase after EGF exposure. p-PAK1 also show reduced basal levels and a delayed accumulation after activation by EGF as compared to control cell lysates. GAPDH was used as loading controls. In C and D.

To explore whether our findings in mice reflect the behavior of oral keratinocytes in humans we decided to explore the consequences of knocking down Rac1 in immortalized normal oral keratinocytes, NOK-SI. We took advantage of the availability of these cells to knockdown Rac1 by using siRNAs targeting the human Rac1 gene (Figure 5A-top). Scratch assay using NOK-SI transfected with Rac1 siRNAs showed a strong inhibition of the migratory capacity of these cells (Figure 5 A-bottom). Interestingly, the reduction of cell migration was also concomitant with a reduced basal and epidermal growth factor (EGF)-induced proliferative capacity of NOK-SI cells as judged by [^3^H] thymidine incorporation (Figure 5B).

Because the Rac/PAK signaling axis has emerged as a key regulatory mechanism controlling epithelial cell migration [28], we investigate the activation status of Rac1 and its downstream targets in the oral keratinocytes NOK-SI and their regulation by growth factors. Using EGF as a mitogen, we detected a remarkable increase in the levels of active Rac1 as early as one min after EGF treatment (Figure 5C), which remained elevated for more than 30 min. We also observed a concomitant activation of JNK, a Rac1 regulated mitogen-activated kinase (MAPK) [29], as judged by the remarkable accumulation of its phosphorylated form, p-JNK (Figure 5C), albeit this was delayed with respect to Rac1 activation, supporting the existence of a complex signaling route from Rac1 to JNK (Figure 5C). There was a rapid activation of PAK1, a direct target of Rac1, although the basal levels of phospho-PAK1 (p-PAK1) were already elevated in these cells hence this activation was more limited. Knock down strategies that reduced Rac1 protein levels in these cells (see above, Figure 5A) resulted in a decreased basal level of active PAK1 and JNK, suggesting that their basal activity is sustained by Rac1 (Figure 5D). Indeed, cells transfected with the control siRNA presented detectable basal levels of p-JNK with a progressively increased activity up to 10 min after EGF stimulation, and p-PAK1 had detectable levels even prior to EGF stimulation. While p-JNK was absent in Rac1 knock down cells, p-PAK1 was barely detectable. Both, however, were stimulated by EGF, albeit it took longer to achieve the same activation levels than in control siRNA transfected cells, suggesting that this growth factor and its receptors may deploy additional, perhaps slower Rac1 independent mechanisms to activate JNK and PAK. We can conclude that Rac1 may control the basal activation status of JNK and PAK1 in oral keratinocyte, and that this small GTPase contributes to growth factor signaling to JNK and PAK1, likely explaining the reduced cell migration and basal and EGF-stimulated proliferation of Rac1-knockdwon epithelial cells.

### *Rac1* excision directly impacts the expression of cytokeratin 6, an activation hyperproliferation-associated marker

**Figure 6:**
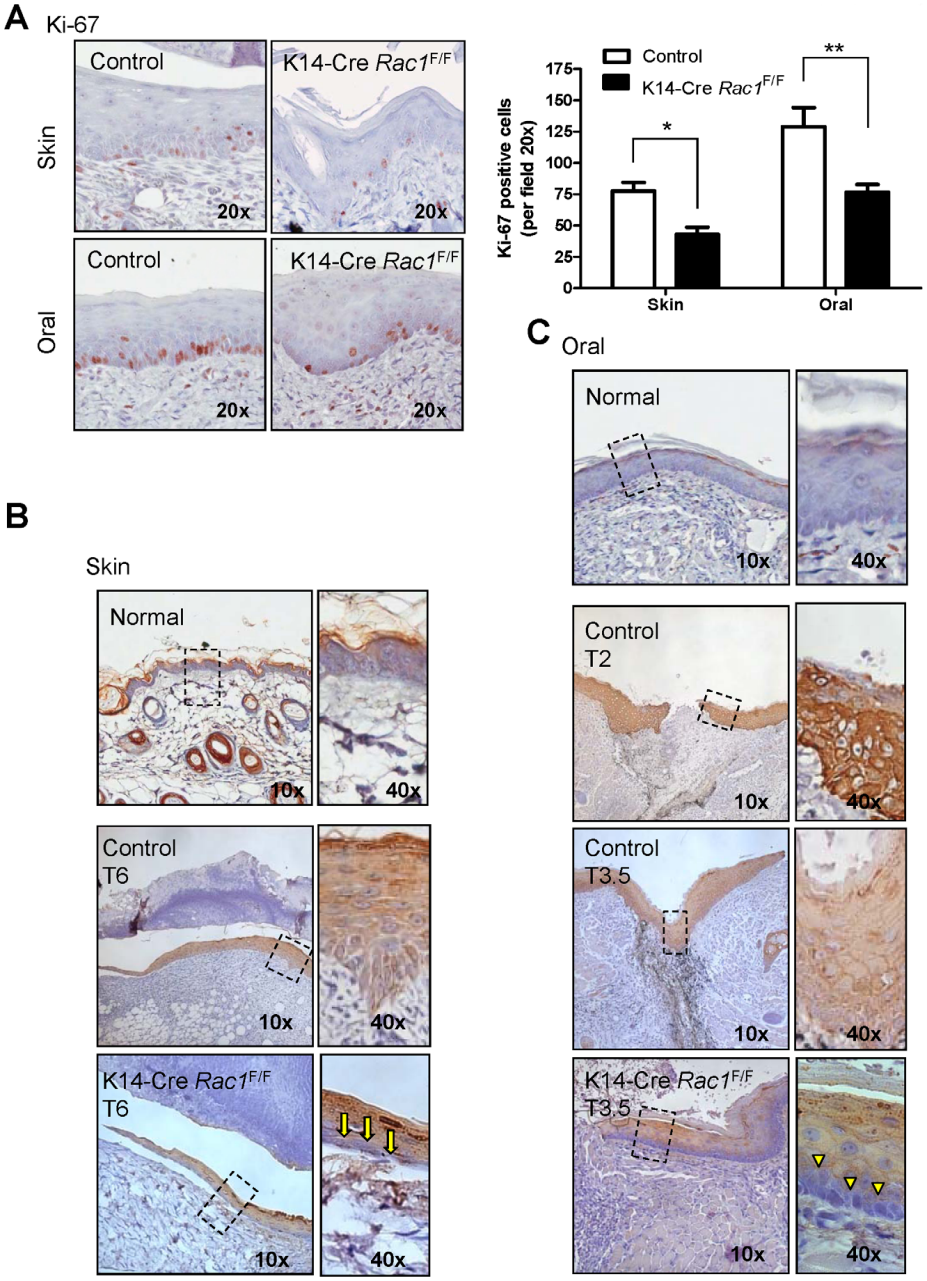
Rac1 excision leads to decreased cell proliferation and limited expression of cytokeratin 6, a stress and growth-related marker, in the skin and oral mucosa after wounding. (A) Impairment of epithelial cell proliferation in the wounded area of the skin and oral mucosa of K14Cre Rac1^F/F^ mice. Sections of epidermis adjacent to wounds healing skin (6 days after injury) and oral mucosa wounds (3.5 days after wounding) from of K14Cre Rac1^F/F^ and control mice were subjected to Ki-67 staining as a marker of cell proliferation. Both anatomical sites of the K14Cre Rac1^F/F^ mice show clear reduction in the proliferation capacity. Bar chart represents the total number of Ki-67 positive cells in the skin and oral cavity of K14Cre Rac1^F/F^ and control mice (**p<0.01, * p<0.05). (B) Normal interfollicular skin does not express detectable levels of cytokeratin 6 (K6). The epithelial tongue adjacent to the wound area from normal skin (control) show remarkable up regulation of K6 in all the epithelial layers after 6 days of injury. K14Cre Rac1^F/F^ mice fail to express K6 in the basal layer of the epithelial tongue from the skin (yellow arrows in the high magnification insert). (C) Normal oral mucosa does not express K6 during homeostasis (Normal). Expression of K6 in the oral mucosa epithelial tongue of control mice is evident at day 2 and 3.5 after injury. Expression pattern of K6 in the oral mucosa wound healing follows the skin pattern. K14Cre Rac1^F/F^ mice show lack of K6 staining in the basal layer of the oral mucosa adjacent to the wound site as depicted (yellow arrowhead).

Although the full characterization of the mechanisms of Rac1 activation and its consequences in oral keratinocytes may be beyond the scope of the present study, these observations prompted us to explore whether the ability to control gene expression programs, in addition to cell migration, may contribute to the healing defects caused by *Rac1* ablation. In this regard, epithelial *Rac1* excision resulted in a reduced proliferative capacity of basal cells from skin and oral mucosa localized next to the epithelial tongue (Figure 6A). On the other hand, JNK is a member of the stress-activated protein kinase family (SAPK), and as such, coordinates gene expression programs in response to cellular stress and inflammation [30]. In particular for keratinocytes, transcriptional factors of the AP-1 and NFκB families, both of which can be controlled by Rac1 [31], are activated during wound healing [32] [33] [34]. As a read-out of this process, we focused on the expression of the keratinocyte stress marker, keratin 6 (K6), which can be induced by hyperproliferative conditions, such as in wound healing, or by conditions that perturb normal keratinocyte function [35] [36] [37]. While constitutive, non-stress related expression of K6 is typical of some stratified epithelia, including the palmo-plantar epidermis and in specialized cells of the hair follicle [38], this keratin is not expressed in normal epidermis and oral mucosa (Figures 6B and 6C - Normal). In contrast, staining tissue sections of K14Cre *Rac1* ^F/F^ mice and their chronological and physiological controls for the stress marker K6 revealed that skin wounds from the control group showed expression of K6 extending from the basal cells up to the cornified layer (Figure 6B-Control), however K14Cre *Rac1* ^F/F^ mice failed to express K6 in the proliferative basal layer (arrows), and detectable levels of K6 was only present in the spinous and cornified layers (Figure 6B- K14Cre *Rac1* ^F/F^), Thus, whereas upon wounding the non-proliferative layers of the epidermis (spinous and granulous layers) from the K14Cre *Rac1* ^F/F^ mice can express K6, the basal layer, which is responsible for cellular proliferation and wound closure, fails to express this stress/hyperproliferative marker, concomitant with a marked decreased in the proliferative potential in the wound area of these mice.

## Discussion

Our understanding on the multiple roles of Rac1 in the regulation of epidermal homeostasis and epidermal stem cell function has substantially improved in recent years. However, in order to fully understand Rac1 function in the skin, we need to dissociate its roles in tissue maintenance during homeostasis from the epidermal response to an acute injury. Functionally, absence of Rac1 from the epidermis did not prevent reconstitution of an IF epidermis after grafting of epidermal stem cells, impacting exclusively on the formation of new HF [14]. Inhibition of Rac1 function or deletion of *Rac1* gene instead leads to decreased keratinocyte proliferation and migration caused by tissue injury [19]. Here we observed that *Rac1* excision from the skin directly impairs the healing of incisional wounds in the skin. Furthermore, although Rac1 ablation in the K14-Cre *Rac1* ^F/F^ mice does not cause any demonstrable effect in the architecture of the stratified epithelium of the oral mucosa [14], the absence of Rac1 in this epithelium which is devoid of HF and their stem cells, leads to a lack of ability to heal wounds, likely due to a decreased migratory, proliferative, or cell differentiating response of the *Rac1*-deficient epithelial cells.

HF stem cells contribute to the healing of dermal wounds, helping IF stem cells to respond to physical injury and reestablish tissue homeostasis [9] [10] [11]. Hence, the lack of Rac1 expression that results in dysfunction of the HF stem cells [14] [17] [18] may explain the reduced ability to heal cutaneous wounds in K14-Cre *Rac1* ^F/F^ mice. However, this may represent an oversimplified perspective, as HF stem cells may not be strictly required for incisional wound healing [13], thus raising the possibility that epithelial Rac1 expression in the IF skin and its resident stem cells may participate in the healing process even if not essential for IF skin development and maintenance [39]. To address this possibility, we chose to focus in the process of healing of tissue injuries in the oral mucosa, which may provide a biologically relevant model system to investigate the re-epithelization of an anatomical site that does not involve the reactivation of HF stem cells. Surprisingly, excision of *Rac1* from the oral mucosa resulted in complete impairment in wound healing. This unexpected result indicates that the stem cell population of the oral mucosa greatly relies in the function of *Rac1* gene to heal open wounds when compared to its more limited role in the skin. Indeed, our results indicate that the epithelial repopulating cells from the oral cavity may function differently from the skin, failing to trigger migratory, proliferative, and differentiating mechanisms in the absence of *Rac1*. This observation was reflected in the inability of the human oral keratinocytes to migrate and proliferate *in vitro* upon Rac1 knock down, and to proliferate and activate gene expression programs resulting in the expression of the stress-related marker K6 in the basal layer of the oral mucosa after the conditional deletion of the *Rac1* gene in mice *in vivo*.

Taken together, we can conclude that Rac1 plays an important role in epithelial cell function, as absence of *Rac1* impairs the response of the epidermis and oral mucosa to an injury. While the proper formation of the HF structures is particularly impaired by *Rac1* excision, the delay in skin wound healing may result from the direct effects of Rac1 on the IF stem cell population or on the ability of their derived transit amplifying cells to proliferate, migrate and terminally differentiate to repopulate the wound area quickly, thereby reestablishing the epithelial barrier function. In the case of oral mucosal injuries, however, ablation of *Rac1* leads to an epithelial proliferation and migration halt. Nonetheless, it is interesting to note that the normal function of the IF skin and oral mucosa can be properly maintained in the absence of Rac1. This suggests that their stem cells are endowed with distinct functional programs. While a Rac1-independent program contributes to skin and oral mucosal development and its homeostasis, a Rac1-dependent program may be strictly required to orchestrate the tissue response to injury and ultimate for wound closure.

Although the precise mechanism by which Rac1 acts warrant further exploration, our emerging results suggest that Rac1 may control the migration of the epithelial stem cells and/or their derived transit amplifying cells, and that this small GTPase may be required for gene expression regulation thereby controlling the proliferation and differentiation of the repopulating epithelial cells and their ability to reconstitute a healthy and functional skin and oral mucosa. Overall, dissociating the normal developmental and homeostatic functions of IF and oral mucosal epithelial stem cells from their role in wound healing, being the latter but not the former Rac1-dependent, may now enable the characterization of the molecular basis of the acute tissue regenerative response of these stem cell populations, thus facilitating the identification of novel molecular-targeted strategies aimed at accelerating wound closure.

## References

[1] Fuchs E (2009). Finding one's niche in the skin.. Cell Stem Cell.

[2] Proksch E, Brandner JM, Jensen JM (2008). The skin: an indispensable barrier.. Exp Dermatol.

[3] Elias PM (1983). Epidermal lipids, barrier function, and desquamation.. J Invest Dermatol.

[4] Elias PM, Choi EH (2005). Interactions among stratum corneum defensive functions.. Exp Dermatol.

[5] Woodley DT, Chen JD, Kim JP, Sarret Y, Iwasaki T (1993). Re-epithelialization. Human keratinocyte locomotion.. Dermatol Clin.

[6] Fuchs E, Nowak JA (2008). Building epithelial tissues from skin stem cells.. Cold Spring Harb Symp Quant Biol.

[7] Singer AJ, Clark RA (1999). Cutaneous wound healing.. N Engl J Med.

[8] Xu X, Lyle S, Liu Y, Solky B, Cotsarelis G (2003). Differential expression of cyclin D1 in the human hair follicle.. Am J Pathol.

[9] Ito M, Liu Y, Yang Z, Nguyen J, Liang F (2005). Stem cells in the hair follicle bulge contribute to wound repair but not to homeostasis of the epidermis.. Nat Med.

[10] Levy V, Lindon C, Harfe BD, Morgan BA (2005). Distinct stem cell populations regenerate the follicle and interfollicular epidermis.. Dev Cell.

[11] Levy V, Lindon C, Zheng Y, Harfe BD, Morgan BA (2007). Epidermal stem cells arise from the hair follicle after wounding.. FASEB J.

[12] Morris RJ, Liu Y, Marles L, Yang Z, Trempus C (2004). Capturing and profiling adult hair follicle stem cells.. Nat Biotechnol.

[13] Langton AK, Herrick SE, Headon DJ (2008). An extended epidermal response heals cutaneous wounds in the absence of a hair follicle stem cell contribution.. J Invest Dermatol.

[14] Castilho RM, Squarize CH, Patel V, Millar SE, Zheng Y (2007). Requirement of Rac1 distinguishes follicular from interfollicular epithelial stem cells.. Oncogene.

[15] Hall A (1998). Rho GTPases and the actin cytoskeleton.. Science.

[16] Braga VM, Betson M, Li X, Lamarche-Vane N (2000). Activation of the small GTPase Rac is sufficient to disrupt cadherin-dependent cell-cell adhesion in normal human keratinocytes.. Mol Biol Cell.

[17] Chrostek A, Wu X, Quondamatteo F, Hu R, Sanecka A (2006). Rac1 is crucial for hair follicle integrity but is not essential for maintenance of the epidermis.. Mol Cell Biol.

[18] Benitah SA, Frye M, Glogauer M, Watt FM (2005). Stem cell depletion through epidermal deletion of Rac1.. Science.

[19] Tscharntke M, Pofahl R, Chrostek-Grashoff A, Smyth N, Niessen C (2007). Impaired epidermal wound healing in vivo upon inhibition or deletion of Rac1.. J Cell Sci.

[20] Heath J, Langton AK, Hammond NL, Overbeek PA, Dixon MJ (2009). Hair follicles are required for optimal growth during lateral skin expansion.. J Invest Dermatol.

[21] Gu Y, Filippi MD, Cancelas JA, Siefring JE, Williams EP (2003). Hematopoietic cell regulation by Rac1 and Rac2 guanosine triphosphatases.. Science.

[22] Andl T, Ahn K, Kairo A, Chu EY, Wine-Lee L (2004). Epithelial Bmpr1a regulates differentiation and proliferation in postnatal hair follicles and is essential for tooth development.. Development.

[23] Castilho RM, Squarize CH, Chodosh LA, Williams BO, Gutkind JS (2009). mTOR mediates Wnt-induced epidermal stem cell exhaustion and aging.. Cell Stem Cell.

[24] Servitja JM, Marinissen MJ, Sodhi A, Bustelo XR, Gutkind JS (2003). Rac1 function is required for Src-induced transformation. Evidence of a role for Tiam1 and Vav2 in Rac activation by Src.. J Biol Chem.

[25] Grafstrom RC, Freshney RI, Freshney MG (2002). Human Oral Epithelium.. Culture of epithelial cells, 2nd ed.

[26] Gutkind JS, Novotny EA, Brann MR, Robbins KC (1991). Muscarinic acetylcholine receptor subtypes as agonist-dependent oncogenes.. Proc Natl Acad Sci U S A.

[27] Bartkova J, Gron B, Dabelsteen E, Bartek J (2003). Cell-cycle regulatory proteins in human wound healing.. Arch Oral Biol.

[28] Etienne-Manneville S, Hall A (2002). Rho GTPases in cell biology.. Nature.

[29] Coso OA, Chiariello M, Yu JC, Teramoto H, Crespo P (1995). The small GTP-binding proteins Rac1 and Cdc42 regulate the activity of the JNK/SAPK signaling pathway.. Cell.

[30] Kaminska B (2005). MAPK signalling pathways as molecular targets for anti-inflammatory therapy–from molecular mechanisms to therapeutic benefits.. Biochim Biophys Acta.

[31] Kwei KA, Finch JS, Ranger-Moore J, Bowden GT (2006). The role of Rac1 in maintaining malignant phenotype of mouse skin tumor cells.. Cancer Lett.

[32] Neub A, Houdek P, Ohnemus U, Moll I, Brandner JM (2007). Biphasic regulation of AP-1 subunits during human epidermal wound healing.. J Invest Dermatol.

[33] Shirai K, Okada Y, Saika S, Senba E, Ohnishi Y (2001). Expression of transcription factor AP-1 in rat lens epithelial cells during wound repair.. Exp Eye Res.

[34] Haas AF, Wong JW, Iwahashi CK, Halliwell B, Cross CE (1998). Redox regulation of wound healing? NF-kappaB activation in cultured human keratinocytes upon wounding and the effect of low energy HeNe irradiation.. Free Radic Biol Med.

[35] Tyner AL, Fuchs E (1986). Evidence for posttranscriptional regulation of the keratins expressed during hyperproliferation and malignant transformation in human epidermis.. J Cell Biol.

[36] Stoler A, Kopan R, Duvic M, Fuchs E (1988). Use of monospecific antisera and cRNA probes to localize the major changes in keratin expression during normal and abnormal epidermal differentiation.. J Cell Biol.

[37] Weiss RA, Eichner R, Sun TT (1984). Monoclonal antibody analysis of keratin expression in epidermal diseases: a 48- and 56-kdalton keratin as molecular markers for hyperproliferative keratinocytes.. J Cell Biol.

[38] Fuchs E (1995). Keratins and the skin.. Annu Rev Cell Dev Biol.

[39] Cotsarelis G (2006). Epithelial stem cells: a folliculocentric view.. J Invest Dermatol.

